# The effects of family structure and function on mental health during China’s transition: a cross-sectional analysis

**DOI:** 10.1186/s12875-017-0630-4

**Published:** 2017-05-05

**Authors:** Yao Cheng, Liuyi Zhang, Fang Wang, Ping Zhang, Beizhu Ye, Yuan Liang

**Affiliations:** Department of Social Medicine and Health Management, School of Public Health, Tongji Medical College, Huazhong University of Science and Technology, Wuhan, China

**Keywords:** Mental health, Family structure, Family function, Urban–rural difference

## Abstract

**Background:**

Social change, intensified by industrialization and globalization, has not only changed people’s work lives but also their personal lives, especially in developing countries. The aim of this study was to provide evidence and recommendations regarding family structure, function, and mental health to actively respond to rapid social change.

**Methods:**

A cross-sectional survey was conducted face-to-face and door-to-door from July 2011 to September 2012 in Hubei Province, central China. Family structure comprised alone, couple, nuclear family, and extended family; family function was measured using the family APGAR (Adaptation, Partnership, Growth, Affection, and Resolve) scale, and mental health was measured using the Chinese version of the 12-item General Health Questionnaire (GHQ-12).

**Results:**

The urban-vs-rural difference of family structure among alone, couple, nuclear family, and extended family was statistically significant (5.21% vs 4.62%; 27.36% vs 13.14%; 33.22% vs 27.74%; 34.20% vs 54.50%, respectively; *p* < 0.0001); and those difference of family function was not statistically significant (8.11 ± 2.13 vs 8.09 ± 2.27, *p* = 0.9372). The general linear regression showed that the effect of family structure on mental health, whether urban or rural, was not significant, however, the effect of family function was significant, especially regarding better family functioning with better mental health.

**Conclusions:**

Combined the effects of family structure and function on mental health, the external form of family (family structure) may not be important; while the internal quality of role (family function) might be key. Improving the residents’ family function would be a priority strategy for family practice with their mental health.

## Background

Social change, intensified by industrialization and globalization, has not only changed people’s work lives but also their personal lives, especially in developing countries [1]. Since the reform and opening-up of China in 1978, and especially in recent decades, Chinese society has undergone unprecedented changes. In the 1982 Chinese Census, the urban population accounted for 20.02% of the total population, increasing to 36.09% by 2000 and to 49.68% in the 2010 Census [2]. Although the growth of the urban population has brought about rapid economic development, it has also resulted in increased pressure on employment, transportation, housing, education, and public health, especially mental health [3]. At the same time, a rapid decline in the rural population combined with the widening urban–rural gap has not only affected agricultural labor, but also the lives of farmers, including their mental health [4]. Another change has been observed in the family structure. This fact is depicted in the reduction of the number of the traditional extended and nuclear families and the increase in the number of alone and couple families [5–7].

Although there are many studies regarding family structure and function and mental health, most focus on special groups such as children, adolescents, immigrants, etc., with very few looking at adult populations [8–12]. However, the family is the basic social unit of the general population and may have an important effect on mental health at all ages. Furthermore, because of intense competition in industrialization and globalization, adult populations face greater pressure in their work and personal lives, and more adults are likely to suffer from mental disorders [13]. Additionally, conflicts between culture and values arising from globalization can not only affect adults’ employment, but also their family life and mental health [14, 15]. Therefore, a study on the adult population’s family structure and function as well as their mental health has important practical significance, especially for developing countries that currently hold a disadvantaged position in global competition. With the world’s largest population, China’s various social problems stemming from industrialization and globalization are very complex. Therefore, China’s experience, whether as a success or failure, may provide important reference values for other developing countries.

To address the above problems, the current study provides an empirical analysis of the effects of family structure and function on mental health via a population-based survey in Hubei Province, central China. The aim of this study is to provide evidence and recommendations regarding family structure, function, and mental health to actively respond to rapid social change.

## Methods

### Participants

Hubei province, where the provincial GDP ranking has been moderate from 2011 to 2014 in China, was determined as the source site of sample. Three cities, Wuhan, Xiaogan and Qianjiang, were selected using purposive sampling, represented respectively of provincial capital city, prefecture-level city and county-level city in Hubei Province. According to Hubei Provincial Bureau of Statistics, the total population (including urban and rural residents) of Wuhan, Xiaogan and Qianjiang city is 8.29, 5.31 and 1.03 million, respectively. In general, urban residents accounted for 54.51% in Hubei province.

**Fig. 1:**
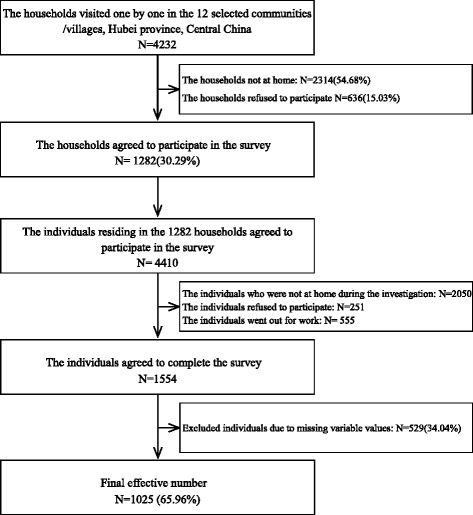
Flowchart for recruitment and response rates to the survey

The current survey was conducted face-to-face and door-to-door from July 2011 to September 2012. The sample size in this study was calculated to be no fewer than 576 individuals by the following determination formulas: *n =* (*t* ^2^ *× π* ^2^ */δ* ^2^) *× deff (*design effect, 1.5*)*, *t =* 1.96, *π =* 0.5, *δ =* 5%, *n ≈* 576, and the total of urban and rural sample is not less than 1152 cases. A method of stratified cluster r sampling was adopted to acquire the study sample. Two communities in urban areas and two administrative villages in rural areas were selected from each city using convenience sampling. The adult residents aged 15 years and older in the selected communities and villages were our targeted samples. Inclusion criteria were the residents aged 15 years and older and willing to participate in the study. Excluded were the residents suffered from terminal illness, diagnosed mental disorder, and not willing to participate. Figure 1 shows the flowchart for recruitment and response rates. Excluding questionnaires with missing data, a total of 1052 (65.96%) valid questionnaires were used in the study.

### Measures

#### Independent variables

Family structure operationalized on the basis of current living arrangement, and comprised the following categories: alone, couple, nuclear family, and extended family. Specifically, alone means a single-person household, and couple means a household with just one couple. It is worth noting the definition of the nuclear family in the current study is used in Chinese way, namely parents living with unmarried children or single parent living with unmarried children, regardless of the age of unmarried children [2, 16]. This definition is in line with the actual situation of Chinese family and cultural traditions. In China, children, even adult children, as long as not married, the vast majority of them are living with their parents. In addition, Chinese extended family means older parents living with their married children and possibly other relatives (representing a traditional Chinese family structure where three or four generations live under the same roof).

Family function was measured using the family APGAR scale, developed by Smilkstein to assess family function on five aspects (Adaptation, Partnership, Growth, Affection, and Resolution) with a three-point scale ranging from 0 (hardly ever) to 2 (almost always) , and it is suitable for use in any ages [17]. The Chinese version of the APGAR was still reliable and repeatable [18, 19], and had relatively good internal consistency (ɑ = 0.75) in the current study. The final family function score is the sum of all five aspects with higher APGAR scores denoting better family function. According to previous studies, an APGAR score of 0–3 is considered to be poor, 4–6 is fair, and 7–10 is good [20, 21].

#### Dependent variable and control variables

The General Health Questionnaire (GHQ) is one of the most common mental health tools developed by D.P. Goldberg in the 1970s [22–24]. We used GHQ-12 to measure individuals’ general psychological healthy status. The Chinese version GHQ-12 was still reliable and repeatable and had relatively good internal consistency (ɑ = 0.76) in the current study. The questionnaire consists of 12 items assessing the severity of a mental problem using a 4-point scale from 0 (never) to 3 (always). Following previous studies, we used a bimodal dataset (0-0-1-1) for scoring that participates indicated low tendency of mental health were defined as 0, or else they were defined as 1. Thus, the possible score range is 0–12. GHQ-12 scores of four and above indicate tendency of mental disorder [25–27]. Sociodemographic variables (i.e., gender, age, education and marital status), self-reported family economic status, smoking, drinking and chronic disease status (e.g., self-reported assessment of a doctor-diagnosed hypertension, diabetes, chronic obstructive pulmonary disease, asthma, and arthritis) were used as control variables.

### Statistical analysis

A descriptive analysis was carried out for the primary variables. The differences in the primary variables between urban and rural residents were tested using Pearson’s *χ* ^*2*^ tests. ANOVA analysis was used to compare GHQ-12 scores and APGAR scores among different family structure, and Bonferroni post-hoc tests were performed. Three generalized linear models were used to examine the independent effects of family structure and function on GHQ-12. Model 1 includes family structure and function only. Gender, age, education, marital status, and self-reported family economic status were added to Model 2, and smoking, drinking, and chronic disease status were added to Model 3.

## Results

Table 1 shows the distribution characteristics of the research variables. There were fewer male participants than female (40.59% vs 59.41%), perhaps because more males were out at work when residents were approached to take part in the survey. Participants aged 60 years or older represented the largest proportion of respondents (41.37%), perhaps again because younger participants were out at work when residents were asked to participate. The proportion of chronic disease status (48.00%) might be related to the proportion of older adults (≥60 years old) of the participants.

**Table 1:** Descriptive Statistics for the primary variables (*n* = 1025)

Variables	Total		Urban(*n* = 614)		Rural(*n* = 411)		*χ* ^*2*^ */F*	*P*
	N/Mean	%/SD	N/Mean	%/SD	N/Mean	%/SD		
Sex								
Male	416	40.59	250	40.72	166	40.39	0.0109	0.9167
Female	609	59.41	364	59.28	245	59.61		
Age (years old)								
15–29	117	11.41	69	11.24	48	11.68	4.8173	0.3066
30–39	115	11.22	73	11.89	42	10.22		
40–49	171	16.68	100	16.29	71	17.27		
50–59	198	19.32	107	17.43	91	22.14		
≥ 60	424	41.37	265	43.16	159	38.69		
Ethnicity								
Han nationality	1016	99.12	606	98.70	410	99.76	3.1730	0.0749
Others	9	0.88	8	1.30	1	0.24		
Religion								
Yes	36	3.51	27	4.40	9	2.19	3.5372	0.0600
No	989	96.49	587	95.60	402	97.81		
Education								
College or more	104	10.15	98	15.96	6	1.46	191.1864	<.0001
Senior high	244	23.80	204	33.22	40	9.73		
Junior high	305	29.76	175	28.50	130	31.63		
Primary or less	372	36.29	137	22.31	235	57.18		
Self-reported economic status								
Good	154	15.02	96	15.64	58	14.11	59.3292	<.0001
Fair	635	61.95	427	69.54	208	50.61		
Bad	236	23.02	91	14.82	145	35.28		
Smoking								
Yes	217	21.17	117	19.06	100	24.33	4.1018	0.0428
No	808	78.83	497	80.94	311	75.67		
Drinking								
Yes	153	14.93	81	13.19	72	17.52	3.6248	0.0569
No	872	85.07	533	86.81	339	82.48		
Chronic disease status								
Yes	492	48.00	297	48.37	195	47.45	0.0845	0.7713
No	533	52.00	317	51.63	216	52.55		
Family structure								
Alone	51	4.98	32	5.21	19	4.62	49.4675	<.0001
Couple	222	21.66	168	27.36	54	13.14		
Nuclear family	318	31.02	204	33.22	114	27.74		
Extended family	434	42.34	210	34.20	224	54.50		
Family function								
(Total APGAR score)	8.10	2.18	8.11	2.13	8.09	2.27	0.01	0.9372
Levels of family function								
Good (APGAR score = 7–10)	810	79.02	487	79.32	323	78.59	0.1951	0.9071
Fair (APGAR score = 4–6)	171	16.68	102	16.61	69	16.79		
Bad (APGAR score = 0–3)	44	4.29	25	4.07	19	4.62		
Mental health								
(GHQ-12 score)	2.92	2.64	2.44	2.34	3.65	2.89	54.81	<.0001
Tendency of mental disorder								
Yes (GHQ-12 score ≥4)	354	34.54	161	26.22	193	46.96	46.7824	<.0001
No (GHQ-12 score <4)	671	65.46	453	73.78	218	53.04		

*Note:* Family function was measured by APGAR scale; mental health was measured by GHQ-12. Total APGAR scores of family function and GHQ-12 scores of mental health between urban and rural populations were compared by ANOVA analysis

The urban-vs-rural difference of family structure among alone, couple, nuclear family, and extended family was statistically significant (5.21% vs 4.62%; 27.36% vs 13.14%; 33.22% vs 27.74%; 34.20% vs 54.50%, respectively; *p* < 0.0001). Family function was generally good (8.08 ± 2.18), and the urban-vs-rural difference was not statistically significant (8.11 ± 2.13vs 8.09 ± 2.27 *p* = 0.9372). GHQ-12 scores were generally low (2.92 ± 2.64), indicating some serious degree of mental illness [], and the urban-vs-rural difference was statistically significant (2.44 ± 2.34 vs 3.65 ± 2.89; *p* < 0.0001). The prevalence of the tendency of mental disorder of rural residents is higher than those of urban residents)(26.22% vs 46.96%; *p* < 0.0001).

Table 2 shows the rural and urban differences between family function and family structure. For urban participants, the family structure “couple” obtained the best function score (8.54 ± 1.88), and “alone” (7.69 ± 2.71) obtained the lowest (*F* = 3.88; *p* = 0.0092). Interestingly, for rural participants, the highest score for family functioning was for those living alone (8.95 ± 1.61), and the lowest was for couples (7.63 ± 2.62). The differences between family function and family structure was not statistically significant (*F* = 1.68; *p* = 0.1712).

**Table 2:** The association of family structure and family function (APGAR)

Family structure	Urban				Rural			
	Mean	SD	*F*	*P*	Mean	SD	*F*	*P*
Alone	7.69	2.71	3.88	0.0092	8.95	1.61	1.68	0.1712
Couple	8.54	1.88			7.63	2.62		
Nuclear family	7.84	2.10			8.10	2.35		
Extended family	8.08	2.19			8.13	2.18		

*Note*: Family structure with urban/rural setting, *F* = 4.43; *P* = 0.0042

Table 3 shows the results of the general linear regression for the effect of the independent variables on mental health (GHQ-12). Model 1 (with family structure and function alone) showed, for both urban and rural residents, that the effect of family structure was not significant. However, the effect of family function was significant, especially regarding better family functioning with better mental health. Similar results were obtained for Models 2 and 3 (adding sociodemographic variables and then smoking, alcohol and chronic disease, respectively). Furthermore, there was little change in *β* values between Models 2 and 3.

**Table 3:** General linear regression for the effect of family structure and function on mental health (GHQ-12)

	Urban			Rural		
	Model 1	Model 2	Model 3	Model 1	Model 2	Model 3
Independent variables						
Family structure (ref = Extended family)						
Alone	−0.36(−1.16,0.44)	−0.41(−1.17,0.35)	−0.45(−1.19,0.29)	0.86(−0.43,2.15)	0.21(−0.99,1.41)	0.19(−0.99,1.37)
Couple	−0.06(−0.49,0.37)	0.12(−0.31,0.55)	0.10(−0.31,0.51)	−0.27(−1.07,0.53)	−0.40(−1.14,0.34)	−0.47(−1.21,0.27)
Nuclear family	0.06(−0.35,0.47)	0.12(−0.29,0.53)	0.16(−0.25,0. 57)	−0.56(−1.17,0.05)	−0.41(−0.998,0.18)	−0.47(−1.04,0.10)
Family function (APGAR)						
	−0.40(−0.48,-0.32)***	−0.33(−0.41,-0.25)***	−0.32 (−0.40,-0.24)***	−0.38(−0.50,-0.26)***	−0.25(−0.35,-0.15)***	−0.25(−0.35,-1.52)***
Control variables						
Gender (ref = Female)						
Male		−0.37(−0.70,-0.04) *	−0.11(−0.28,0.50)		−0.54(−1.07,-0.01) *	−0.50(−1.17,0. 17)
Age(ref = ≥60 years old)						
15-29		0.35(−0.26,0.96)	0.83(0.18,1.48)*		−0.83(−1.81,0.15)	−0.38(−1.40,0.64)
30-39		0.14(−0.43,0.71)	0.64(0.03,1.25)*		−0.14(−1,12,0.84)	0.32(−0.70,1.34)
40-49		0.44(−0.11,0.99)	0.89(0.32,1.46)**		−0.51(−1.29,0.27)	−0.15(−0.95,0.65)
50-59		0.13(−0.36,0.62)	0.40(−0.09,0.89)		0.52(−0.17,1.21)	0.67(−0.02,1.36)
Education level (ref = Primary or less)						
Senior high or more		−0.55(−1.02,-0.08)*	−0.57(−1.04,-0.10)**		−0.94(−1.84,-0.04) *	−0.87(−1.75,0.01)
Junior high		0.14(−0.33,0.61)	−0.03(−0.50,0.44)		−0.14(−0.79,0.51)	−0.15(−0.78,0.48)
Self-perceived economic status(ref = Bad)						
Good/Fair		−2.03(−2.50,-1.56)***	−1.93(−2.38,-1.48)***		−2.29(−2.80,-1.78)***	−2.15(−2.66,-1.64)***
Tobacco smoking(ref = No)						
Yes			−0.36(−0.85,0.13)			0.35(−0.38,1.08)
Alcohol drinking(ref = No)						
Yes			−0.26(−0.79,0.27)		d	−0.59(−1.32,0.14)
Chronic disease status(ref = No)						
Yes			0.82(0.45,1.19)***			0.81(0.26,1.36)**
*R* ^*2*^	0.14	0.27	0.30	0.10	0.30	0.32

Note: Model 1 only use family structure and function; then Model 2, age, gender, education level, self-perceived economic status were added; then Model 3, tobacco smoking, alcohol drinking and chronic disease status were added

*p* < 0.05*, *p* < 0.01**, *p* < 0.001***

## Discussion

This study provides an evidence for the effects of family structure and function on mental health of adult population during China’s transition. Overall, our findings suggest that the urban–rural difference of family structure was statistically significant, and those of family function was not statistically significant; and the effect of family structure on mental health, whether urban or rural, was not significant, however, those of family function was significant.

### Family structure and function

The change in family structure may be related to two aspects. One concerns an internal cause; that is, familial changes brought about for personal reasons. For example, older family members (especially older parents) may choose to live alone because they do not want to burden their children or grandchildren [28]. Meanwhile, younger family members (especially adult children living in the city) cannot afford to preserve the traditional extended family lifestyle, and may not have the ability to support their parents (because of rising house prices, job instability, and a lack of income security) [29, 30]. A further reason, external to the family situation, is conflict between culture and values. The past 20–30 years, with the advent of globalization and fierce market competition, have marked the rise of the ego and money worship, with a decrease in altruism and spiritualism [31]. Such changes will not only affect people’s work and family lives, but also create interpersonal tensions and work–family conflict, as well as impacting on mental health [32].

Because of the difference in measurement tools, evaluation methods and participants, comparative analysis of family function is difficult among different studies. In the current study, overall, family function is good (the proportion deemed “poor” only accounted for 3.85% in urban areas and 4.67% in rural areas, and the urban–rural difference was not statistically significant). In contrast with changes in family structure, changes in family function are not obvious. While family structure has changed, family function appears to have remained relatively stable, raising two questions: Why has family function not changed along with family structure? Will a change to family structure lead to a change in family function? An example of a pathological phenomenon may help us analyze this problem. Cirrhosis of the liver can be caused by a virus, but the liver may still function well (because of physiological compensation) when the disease is in its early stages or is relatively mild. Similarly, the current changes in family structure may be at an early stage, and so family function may still be good. Long-term changes in family structure could lead to changes in family function [33], so we need to take measures as early as possible to ensure that healthy functioning continues. This is very important during the transition period, especially for developing countries that are currently at a disadvantage in global competition.

### Mental health

This study showed that the overall proportion of mental disorders is relatively high, and higher for those in rural areas than urban areas (46.89% vs 26.92%). This result reveals the severity and breadth of mental health with social change, especially for rural residents [34–38]. One reason for this result may be the economic conditions in urban and rural areas [39]. The percentage of self-reported economic status deemed “bad” among urban and rural residents is 14.89 and 35.79%, respectively. Poor economic conditions will increase burdens not only on living conditions and social status, but also on other factors including health and children’s education. A further reason for this result is the reduction of the rural population and the growth of the urban–rural gap. Combined with the rise of egoism and money worship, this could result in greater mental ill health.

### The effect of family structure and function on mental health

Our multivariate analysis showed that better family functioning was associated with better mental status. Previous research on special populations such as children, adolescents, and immigrants showed that family function had a protective effect on mental health [8–10, 40]. The present study confined such protection effects to the adult population. It is worth noting that the impact of family structure on mental health, whether urban or rural, was not statistically significant. When looking at the combined effects of family structure and function, the external form of family (family structure) may not be important, while the internal qualify of role (family function) might be key.

With irreversible globalization, long-term changes in family structure may affect family function, thereby affecting the mental health of family members. Whether traditional culture or social reality, China’s traditional family functions cannot be replaced. Chinese families not only provide the functions of production, education, maternity, and pension, but also that of psychological comfort and support, which involves the health of all family members, as well as the stability and development of society as a whole. Faced with social change and health problems, individuals and families may be powerless. Thus, all facets of society should work together, which is particularly important for developing countries struggling to advance under globalization.

### Limitations

First, the response rate in this study was relatively low, which could produce selection bias. With regard to the employment status of the adult population, our door-to-door survey in urban areas was conducted during weekends (Saturday and Sunday), rather than during the week (Monday to Friday) when adult participants were more likely to be out at work. However, because of the residents’ reluctance to participate in the survey, especially in urban areas, it was very difficult for us to improve the response rate. Moreover, large families are more likely to left family member stay at home, and residents’ reluctance to participate from same families might be relatively lower, so their participation rate would be high. Low response rates as well as the corresponding selection bias may be a reflection of social change on family structure and function. Second, as mentioned above, we were unable to determine the proportion of urban residents that had originated from rural areas in the past 10–20 years, which would affect the depth of the current study. Third, our sample was confined to three cities in a central province in China, which may reduce the generalizability of our findings. Forth, this study was a cross-sectional design, meaning that the statistical analysis neglects the term of time in the inferences. Thus, the observed associations could not be considered causal, and there is a high possibility that these associations are false positives. The findings should be examined and replicated in a study with prospective design to examine the associations. Also, the observed effect of family structure and function on mental health could be caused by residual confounding. Additional, we applied linear models to examine this association taking into account some factors (age, gender, education, marital status, and family economic status), other factors not considered might act as confounders in this study.

## Conclusion

As the world’s largest developing country, the family structure and function as well as their impact on mental health are particularly noteworthy during China’s transition. Combined the effects of family structure and function, the external form of family (family structure) may not be important, the internal quality of role (family function) might be the key. Improving the residents’ family function would be a priority strategy for family practice with their mental health.

## Abbreviations

APGAR: Adaptation Partnership Growth Affection Resolve
GHQ-12: the 12-item General Health Questionnaire
